# Supplementary material for: Trapped ion mobility spectrometry and PASEF enable in-depth lipidomics from minimal sample amounts
Source: Nat Commun. 2020 Jan 16;11:331. doi: 10.1038/s41467-019-14044-x (PMC6965134; doi:10.1038/s41467-019-14044-x)
Supplement: Supplementary file 1 — Supplementary Information [file 41467_2019_14044_MOESM1_ESM.pdf]

Supplementary Information

Trapped ion mobility spectrometry and PASEF enable in-depth lipidomics from minimal sample amounts

Catherine G. Vasilopoulou[1], Karolina Sulek[2], Andreas-David Brunner[1], Ningombam Sanjib Meitei[3], Ulrike Schweiger-Hufnagel[4], Sven W. Meyer[4], Aiko Barsch[4], Matthias Mann[1,2], Florian Meier[1]

[1]Max-Planck Institute of Biochemistry, Martinsried, Germany

[2]NNF Center for Protein Research, Copenhagen, Denmark

[3]PREMIER Biosoft, Indore, India

[4]Bruker Daltonik GmbH, Bremen, Germany

Supplementary Figure 1

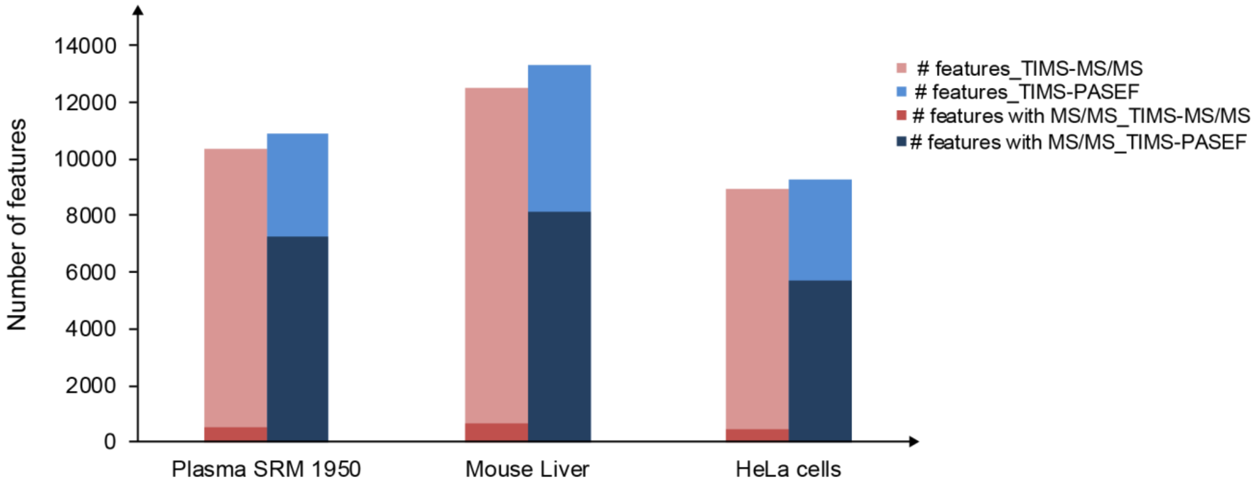

Supplementary Fig. 1. **Evaluation of PASEF in lipidomics.** Total number of 4D features extracted from 30 min runs of human plasma (n=5), mouse liver (n=5) and human cancer cells (n=5) in negative mode without (TIMS-MS/MS, red) and with PASEF (TIMS-PASEF, blue). The fraction of features assigned to MS/MS spectra is indicated by a darker color.

Supplementary Figure 2

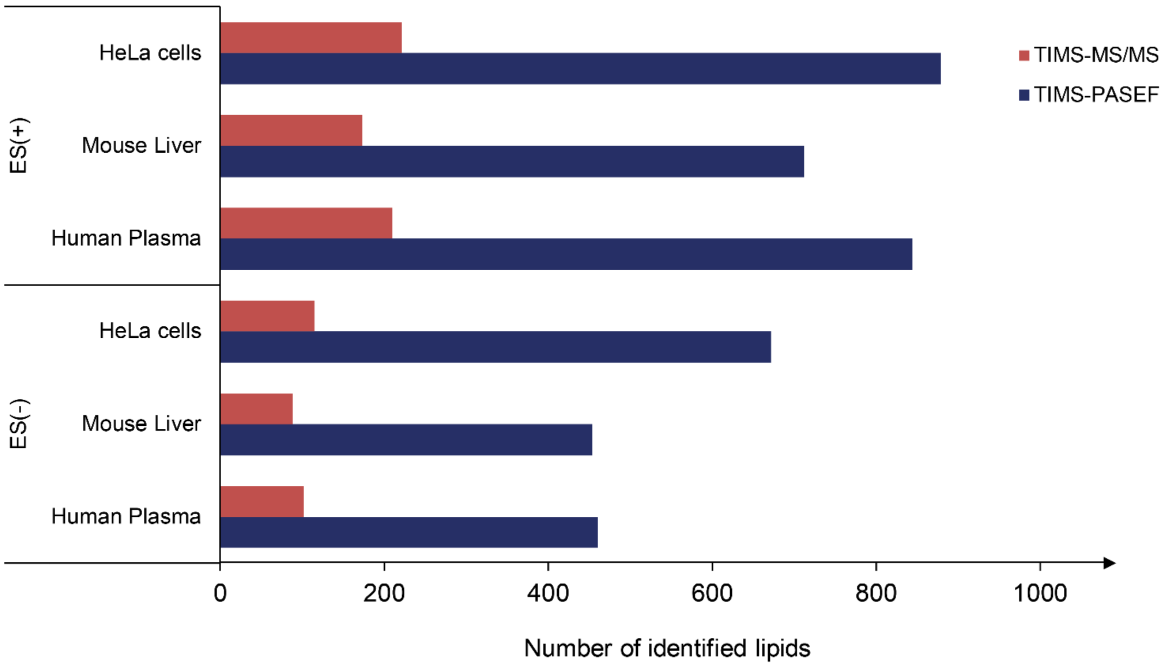

Supplementary Fig. 2. Number of identified lipids in short LC-MS runs (30min). Total number of identified lipids in human plasma, mouse liver and human cancer cell extracts in both positive and negative ionization modes without (TIMS-MS/MS, red) and with PASEF (TIMS-PASEF, blue).

Supplementary Figure 3

a.

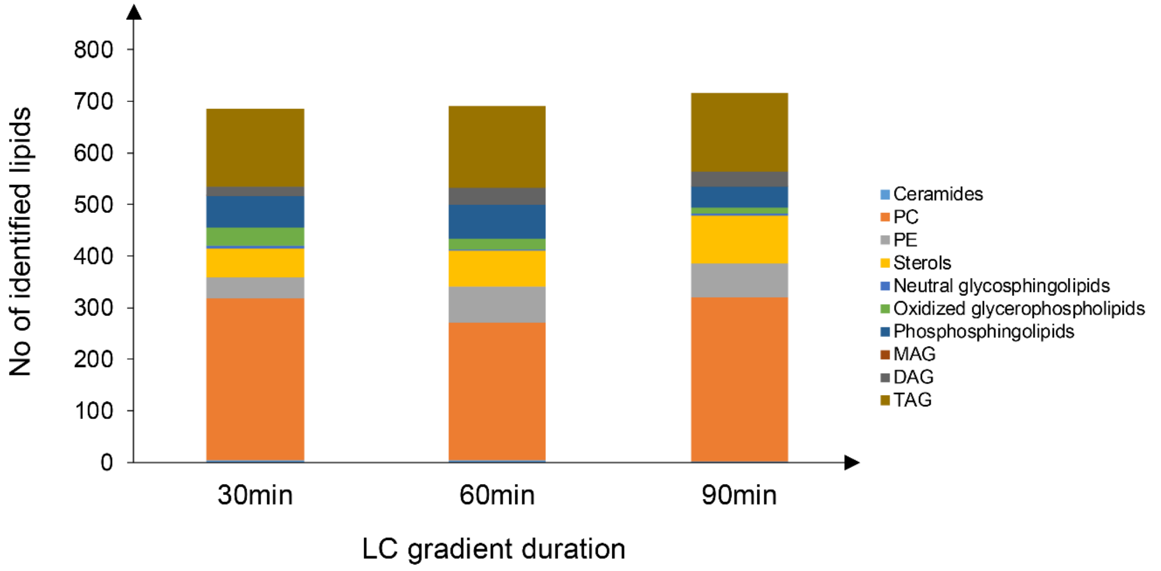

b.

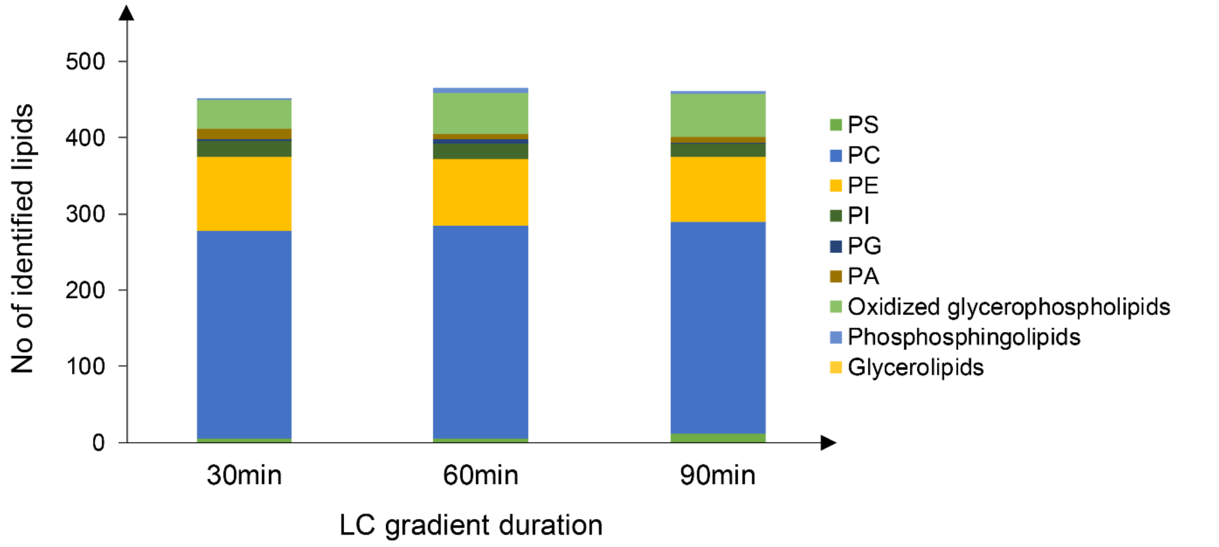

Supplementary Fig. 3. Number of identified lipids in different LC gradient durations. a,b, Number of identified lipids from various lipid classes in 30, 60 and 90 min nanoflow LC-MS runs of human plasma in **a,** positive and **b,** negative ionization mode.

PC= Phospatidylcholine, PE=Phospatidylethanolamine, PA= Phosphatidic acid, PI= Phospatidylinositol, PG=Phospatidylglycerol, PS=Phospatidylserine, MAG=Monoacylglycerol, DAG=Diacylglycerol, TAG=Triacylglycerol.

Supplementary Figure 4

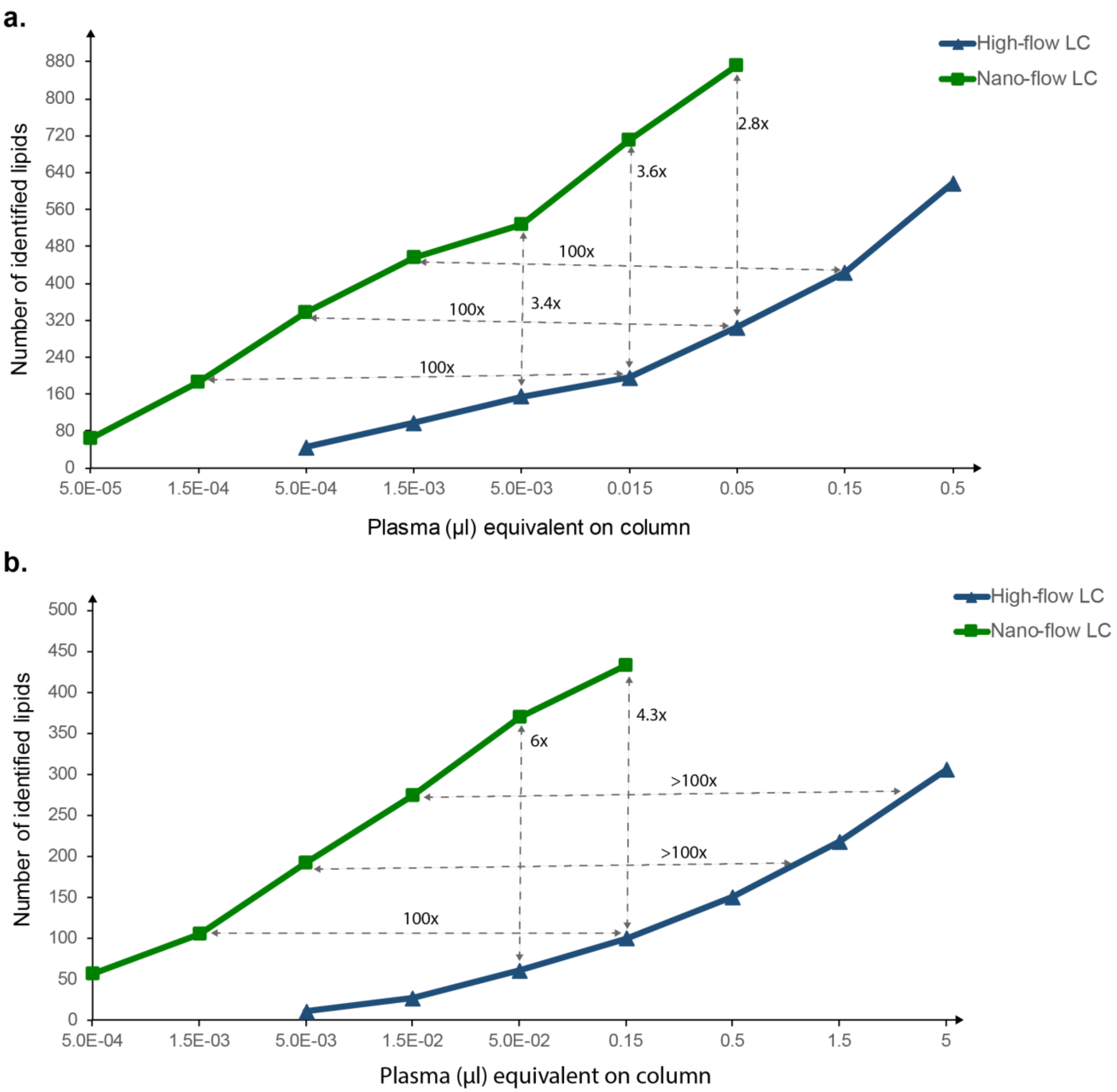

Supplementary Fig. 4. Number of identified lipids in various concentrations of human plasma SRM 1950 lipid extract with high-flow and nanoflow LC-MS setups. a,b, Number of identified lipids from various lipid classes as a function of the sample amount on column using a high-flow (blue) and a nanoflow (green) LC system in **a,** positive and **b,** negative ionization mode.

Supplementary Figure 5

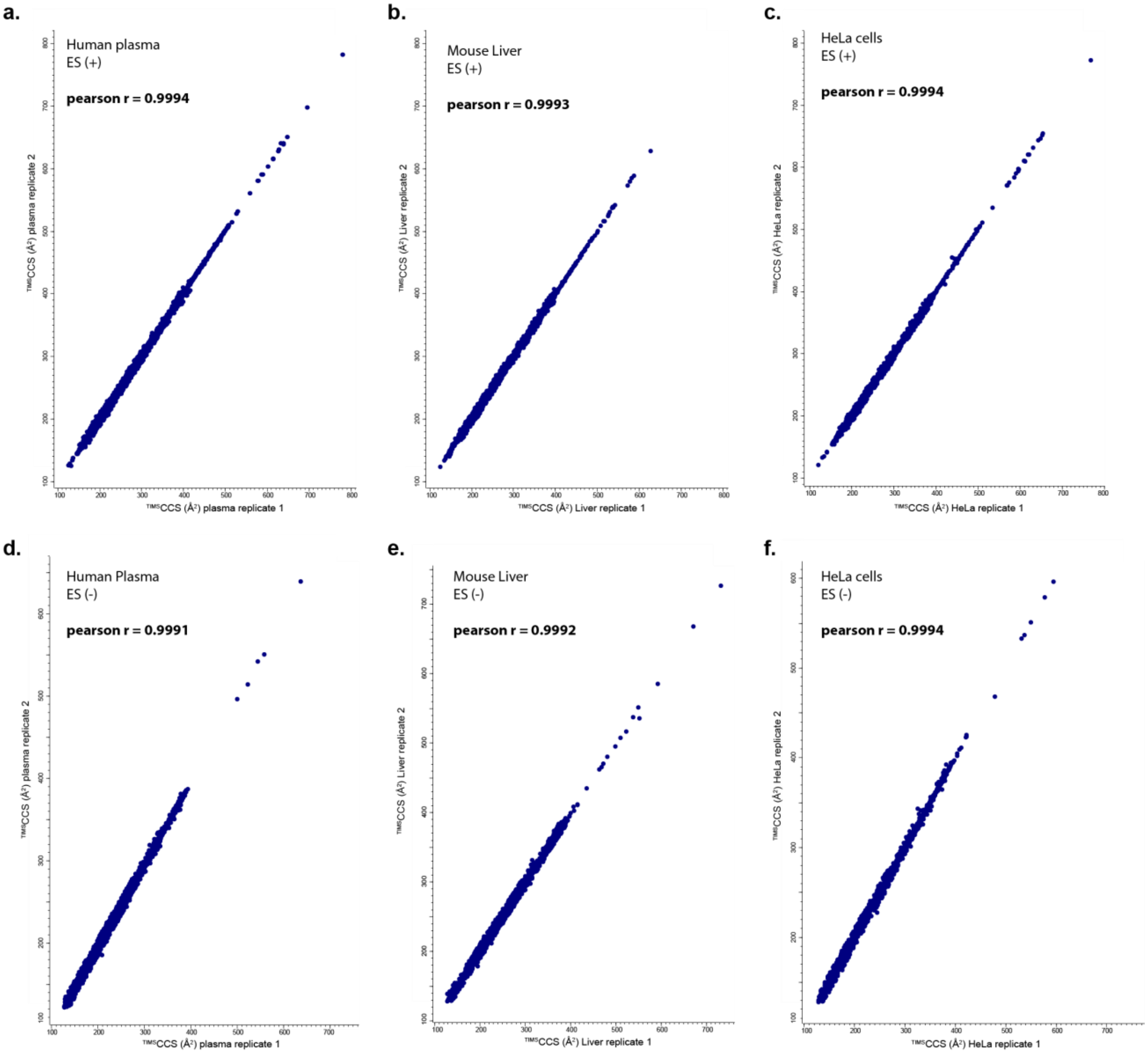

Supplementary Fig. 5. Precise measurements of ^{TIMS}CCS values in lipid extracts from complex biological samples. Pearson correlation of ^{TIMS}CCS values of all 4D features detected in two replicate injections of **a,** human plasma extract in positive mode, **b,** mouse liver extract in positive mode, **c,** HeLa cells extract in positive mode, **d,** human plasma extract in negative mode, **e,** mouse liver extract in negative mode, and **f,** Hela cells extract in negative mode.

Supplementary Figure 6

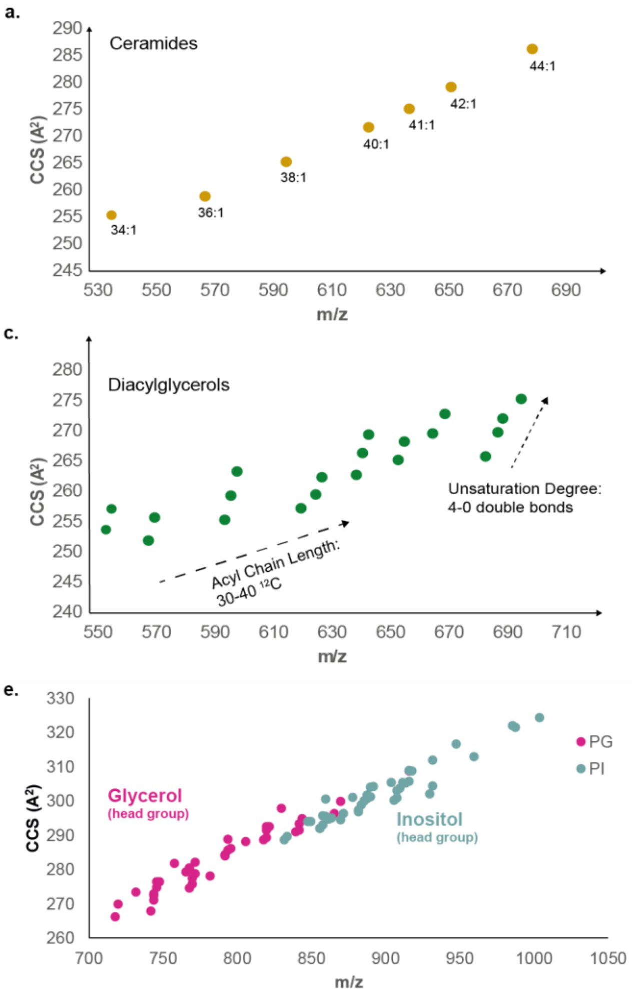

Supplementary Fig. 6. Investigation of the effect of lipid composition on the ^{TIMS}CCS values. a-e, Influence of acyl chain length and unsaturation degree on the CCS vs *m/z* distribution for **a,** Ceramides, **b,** Phosphatidylcholines, **c,** Diacylglycerols, **d,** Triacylglycerols, and **e,** effect of head group (glycerol and inositol of glycerophospholipids).
