# Supplementary material for: Trapped ion mobility spectrometry and PASEF enable in-depth lipidomics from minimal sample amounts
Source: Nat Commun. 2020 Jan 16;11:331. doi: 10.1038/s41467-019-14044-x (PMC6965134; doi:10.1038/s41467-019-14044-x)
Supplement: Supplementary file 2 — Description of Additional Supplementary Files [file 41467_2019_14044_MOESM2_ESM.docx]

File Name: Supplementary Data 1

Description: Retention times (s) of identified lipids in five replicate injections of plasma SRM 1950 extract using the nano- and high-flow LC systems.

File Name: Supplementary Data 2

Description: Lipids identified in human NIST SRM 1950 plasma extract. Lipids are grouped by the lipid class (column A). Further information such as retention time, *m/z*, CCS values, categorization, delta mass, number of double bonds, LSI ID, LIPID MAPS ID, matched fragment ions etc. are provided.

File Name: Supplementary Data 3

Description: Lipids identified in mouse liver extract. Lipids are grouped by the lipid class (column A). Further information such as retention time, *m/z*, CCS values, categorization, delta mass, number of double bonds, LSI ID, LIPID MAPS ID, matched fragment ions etc. are provided.

File Name: Supplementary Data 4

Description: Lipids identified in human HeLa cells extract. Lipids are grouped by the lipid class (column A). Further information such as retention time, *m/z*, CCS values, categorization, delta mass, number of double bonds, LSI ID, LIPID MAPS ID, matched fragment ions etc. are provided.

File Name: Supplementary Data 5

Description: Quantification (Intensity) values for lipids identified in human NIST SRM 1950 plasma extract. In the left table (orange), the number of ‘zero’ or missing values has been calculated (Fig.3b). In the right table, (green), the coefficient of variation (CV) has been calculated (Fig.3c).

File Name: Supplementary Data 6

Description: Lipids identified in the Quehenberger et al. study. TRUE indicates the commonly identified lipids based on the short name annotation while FALSE indicates lipids uniquely identified in Quehenberger et al. study.

File Name: Supplementary Data 7

Description: Lipids identified in the Bowden et al. study. TRUE indicates the commonly identified lipids based on the short name annotation while FALSE indicates lipids uniquely identified in Bowden et al*.* study.

File Name: Supplementary Data 8

Description: ^TIMS^CCS values of 22 lipid species identified in five replicate injections of the Differential Ion Mobility Mix on four timsTOF Pro instruments (Munich and Bremen, Germany). Relative intra-instrument, intra-laboratory and inter-laboratory variations are provided.

File Name: Supplementary Data 9

Description: ^TIMS^CCS values of MS features detected in each of the three sample types in both ionization modes on a single timsTOF Pro instrument (Munich, tims1).

File Name: Supplementary Data 10

Description: ^TIMS^CCS values of identified lipids in five replicate injections of plasma SRM 1950 extract in positive mode.

File Name: Supplementary Data 11

Description: ^TIMS^CCS values commonly identified in five replicates of plasma SRM 1950 extract across four timsTOF Pro instruments (Munich and Bremen, Germany). Relative intra-instrument, intra-laboratory and inter-laboratory variations are provided.

File Name: Supplementary Data 12

Description: CCS values of commonly identified lipids (based on short name annotation) in our study and reported by the Zhu and McLean laboratories.

File Name: Supplementary Data 13

Description: Experimental and machine-learning predicted CCS values for 2,126 lipids identified in positive mode in all three biological samples (Human plasma, mouse liver, HeLa cells).

File Name: Supplementary Data 14

Description: 4D lipid dataset comprising CCS values of 1,856 unique lipids identified in all three sample types in positive mode, representing the four major lipid categories and 15 lipid classes. Further information such as retention time, *m/z*, ion mobility and CCS values, categorization, delta mass, number of double bonds, LSI ID, LIPID MAPS ID, matched fragment ions etc. are provided.
