# Supplementary material for: Trapped ion mobility spectrometry and PASEF enable in-depth lipidomics from minimal sample amounts
Source: Nat Commun. 2020 Jan 16;11:331. doi: 10.1038/s41467-019-14044-x (PMC6965134; doi:10.1038/s41467-019-14044-x)
Supplement: Supplementary file 17 — Reporting Summary [file 41467_2019_14044_MOESM17_ESM.pdf]

nature research

Corresponding author(s): Mann, Meier

Last updated by author(s): Dec 11, 2019

Reporting Summary

Nature Research wishes to improve the reproducibility of the work that we publish. This form provides structure for consistency and transparency in reporting. For further information on Nature Research policies, see Authors & Referees and the Editorial Policy Checklist .

Statistics

For all statistical analyses, confirm that the following items are present in the figure legend, table legend, main text, or Methods section.

n/a	Confirmed	
☐	☒	The exact sample size (*n*) for each experimental group/condition, given as a discrete number and unit of measurement
☐	☒	A statement on whether measurements were taken from distinct samples or whether the same sample was measured repeatedly
☒	☐	The statistical test(s) used AND whether they are one- or two-sided<br>*Only common tests should be described solely by name; describe more complex techniques in the Methods section.*
☒	☐	A description of all covariates tested
☒	☐	A description of any assumptions or corrections, such as tests of normality and adjustment for multiple comparisons
☐	☒	A full description of the statistical parameters including central tendency (e.g. means) or other basic estimates (e.g. regression coefficient) AND variation (e.g. standard deviation) or associated estimates of uncertainty (e.g. confidence intervals)
☒	☐	For null hypothesis testing, the test statistic (e.g. *F*, *t*, *r*) with confidence intervals, effect sizes, degrees of freedom and *P* value noted<br>*Give P values as exact values whenever suitable.*
☒	☐	For Bayesian analysis, information on the choice of priors and Markov chain Monte Carlo settings
☒	☐	For hierarchical and complex designs, identification of the appropriate level for tests and full reporting of outcomes
☒	☐	Estimates of effect sizes (e.g. Cohen's *d*, Pearson's *r*), indicating how they were calculated

Our web collection on statistics for biologists contains articles on many of the points above.

Software and code

Policy information about availability of computer code

Data collection	MS raw data were recorded with Bruker otofControl version 6.0
Data analysis	MS raw data were analyzed with Bruker MetaboScape v5.0 (feature detection and quantification). Lipid MS/MS spectra were annotated with Premier Biosoft SimLipid v6.05. Data filtering and visualization was performed in Microsoft Excel 2016, Python 3 (Jupyter notebook) and Perseus (v1.6.0.8). MS metadata were extracted from the .tdf files using an SQLite database viewer (SQLite Manager v3.10.1).

For manuscripts utilizing custom algorithms or software that are central to the research but not yet described in published literature, software must be made available to editors/reviewers. We strongly encourage code deposition in a community repository (e.g. GitHub). See the Nature Research guidelines for submitting code & software for further information.

Data

Policy information about availability of data

All manuscripts must include a data availability statement. This statement should provide the following information, where applicable:

- Accession codes, unique identifiers, or web links for publicly available datasets
- A list of figures that have associated raw data
- A description of any restrictions on data availability

The mass spectrometry raw files have been uploaded to the MASS Spectrometry Interactive Virtual Environment (MassIVE) and are accessible via the identifier MSV000083858 [https://dx.doi.org/10.25345/C51063].

Field-specific reporting

Please select the one below that is the best fit for your research. If you are not sure, read the appropriate sections before making your selection.

Life sciences study design

All studies must disclose on these points even when the disclosure is negative.

Sample size	No sample-size calculation was performed. Sample size was determined to reflect technical reproducibility in triplicate or more repeated injections and assess differences due to acquisition parameters.
Data exclusions	No data were excluded from the analyses.
Replication	All attempts at replication were successful.
Randomization	Randomization was not relevant to this study as is focuses on technical variations rather than biological conclusions.
Blinding	Blinding was not relevant to this study as is focuses on technical variations rather than biological conclusions.

Reporting for specific materials, systems and methods

We require information from authors about some types of materials, experimental systems and methods used in many studies. Here, indicate whether each material, system or method listed is relevant to your study. If you are not sure if a list item applies to your research, read the appropriate section before selecting a response.

Materials & experimental systems

n/a	Involved in the study	
☒	☐	Antibodies
☐	☒	Eukaryotic cell lines
☒	☐	Palaeontology
☐	☒	Animals and other organisms
☒	☐	Human research participants
☒	☐	Clinical data

Methods

n/a	Involved in the study	
☒	☐	ChIP-seq
☒	☐	Flow cytometry
☒	☐	MRI-based neuroimaging

Eukaryotic cell lines

Policy information about cell lines

Cell line source(s)	The HeLa cell line was obtained from DSMZ, number ACC 57.
Authentication	The cell line has not been authenticated in the course of this study.
Mycoplasma contamination	The cell line was tested negative for mycoplasma contamination.
Commonly misidentified lines (See ICLAC register)	No commonly misidentified cell lines were used.

Animals and other organisms

Policy information about studies involving animals; ARRIVE guidelines recommended for reporting animal research

Laboratory animals	mus musculus, C57BL/6, male
Wild animals	The study did not involve wild animals.
Field-collected samples	The study did not involve samples collected from the field.
Ethics oversight	Animal experiments were performed in compliance with the ethical and institutional regulations of the Max Planck Institute of Biochemistry for animal testing and research, and have been approved by the government agencies of Upper Bavaria.

Note that full information on the approval of the study protocol must also be provided in the manuscript.
